# Effectiveness of Guided and Unguided Low-Intensity Internet Interventions for Adult Alcohol Misuse: A Meta-Analysis

**DOI:** 10.1371/journal.pone.0099912

**Published:** 2014-06-17

**Authors:** Heleen Riper, Matthijs Blankers, Hana Hadiwijaya, John Cunningham, Stella Clarke, Reinout Wiers, David Ebert, Pim Cuijpers

**Affiliations:** 1 Department of Clinical Psychology and EMGO Institute, VU University Amsterdam, Amsterdam, Netherlands; 2 Division Health Trainings online, EU innovation Incubator, Leuphana University, Lüneburg, Germany; 3 Arkin Mental Health Care, Amsterdam, the Netherlands; 4 Trimbos Institute, Netherlands Institute of Mental Health and Addiction, Utrecht, The Netherlands; 5 Amsterdam Institute for Addiction Research, Department of Psychiatry, Academic Medical Centre, University of Amsterdam, the Netherlands; 6 Centre for Mental Health Research, the Australian National University, Canberra, Australia; 7 Centre for Addiction and Mental Health, Toronto, Canada; 8 NHS Fife, Edinburgh, Scotland, United Kingdom; 9 Addiction, Development and Psychopathology (ADAPT) lab, Department of Psychology, University of Amsterdam, Amsterdam, The Netherlands; 10 Division for Clinical Psychology and Psychotherapy, Department for Psychology, Philipp’s University Marburg, Marburg, Germany; 11 TS Social and Behavioral Sciences, Department of Developmental Psychology, Tilburg University, Tilburg, the Netherlands; Institute of Psychiatry, United Kingdom

## Abstract

**Background:**

Alcohol misuse ranks within the top ten health conditions with the highest global burden of disease. Low-intensity, Internet interventions for curbing adult alcohol misuse have been shown effective. Few meta-analyses have been carried out, however, and they have involved small numbers of studies, lacked indicators of drinking within low risk guidelines, and examined the effectiveness of *unguided* self-help only. We therefore conducted a more thorough meta-analysis that included both *guided* and *unguided* interventions.

**Methods:**

Systematic literature searches were performed up to September 2013. Primary outcome was the mean level of alcohol consumption and drinking within low risk guidelines for alcohol consumption at post-treatment.

**Findings:**

We selected 16 randomised controlled trials (with 23 comparisons and 5,612 participants) for inclusion. Results, showed a small but significant overall effect size in favour of Internet interventions (*g* = 0.20, 95% CI: 0.13–0.27, *p*<.001). Participants in Internet interventions drunk on average 22 grams of ethanol less than controls and were significantly more likely to be adhering to low-risk drinking guidelines at post-treatment (RD 0.13, 95% CI: 0.09–0.17, *p*<.001). Subgroup analyses revealed no significant differences in potential moderators for the outcome of alcohol consumption, although there was a near-significant difference between comparisons with waitlist control and those with assessment-only or alcohol information control conditions (*p* = .056).

**Conclusions:**

Internet interventions are effective in reducing adult alcohol consumption and inducing alcohol users to adhere to guidelines for low-risk drinking. This effect is small but from a public health point of view this may warrant large scale implementation at low cost of Internet interventions for adult alcohol misuse. Moderator analyses with sufficient power are, however, needed in order to assess the robustness of these overall results and to assess whether these interventions may impact on subgroups with different levels of success.

## Introduction

The *Global Burden of Disease Study 2010* from the World Health Organization has documented a growing health burden from Alcohol Use Disorders (AUDs) over the past two decades among adults in both developed and developing societies [1], [2]. The health burden increases further if the whole spectrum of alcohol misuse [3] is taken into account, whereby people consume alcohol in excess of the low-risk drinking guidelines but do not meet AUD criteria [1], [4], [5]. They include people who engage in *hazardous alcohol use* – and who may thereby develop physical, psychological and social problems in the short term and alcohol dependency and serious or fatal illness later on – or *harmful alcohol use*, meaning that they are already experiencing such problems [4]. This mounting health burden is due not just to population growth or ageing, but also to an absolute increase in alcohol consumption by adults [1], [6]. It affects both traditional groups of drinkers and newer groups such as women [7] and the elderly [8]. The risks of problem drinking multiply with every increment in alcohol consumption or every heavier drinking pattern [9], [10]. Estimates reveal that eight out of ten adults who drink too much alcohol lack professional help; if they do receive it, that is often at a very late stage in their drinking career [11]. Studies also show that whilst a high number of people who misuse alcohol do desire help, they prefer it outside conventional health care settings [12]. Public health strategies to curb alcohol misuse therefore have considerable room for improvement. One particular enhancement could lie in an expanded availability of brief, low-intensity Internet interventions for use both in and beyond the primary care sector [13], [14].

Conventional (that is, non-Internet) Screening and Brief Interventions (SBIs) have been evaluated mostly in their delivery to non–alcohol-dependent, non–treatment-seeking adults whose alcohol misuse was identified in opportunistic health screening in primary care. Numerous meta-analyses and systematic reviews have convincingly shown the clinical effectiveness of SBIs in reducing alcohol consumption in comparison to non-intervention controls. The results of all these studies have recently been synthesised in studies by Jonas and colleagues [15], [16], and these in turn by Moyer in her 2013 update to the United States Preventive Services Task Force’s earlier recommendation statement on screening and brief interventions in primary care for alcohol misuse [3]. Overall, the results show significant effect sizes in the small to moderate range for low-intensity interventions in comparison to control conditions in terms of average decreases in alcohol consumption [3], [15], [16]. Cost-effectiveness studies, albeit limited in number, also report promising results [17]–[19]. Effectiveness studies on the reduction of alcohol consumption through SBIs provided in other settings such as general hospitals, emergency departments or work environments have been inconclusive [20]–[23].

Providing Internet self-help interventions both in primary care and directly to people in the community appears a promising strategy to overcome the gap between the high number of people that misuse alcohol and the low number that actually receive or seek help in primary care [24]. Studies on web-based self-help interventions for adult problem drinking show that (1) the interventions are mostly of an unguided nature and are delivered as stand-alone procedures directly to participants in the community, and to a far lesser extent via primary care, clinical or employment settings [25], [26]; (2) they largely reach first-time help seekers (with rates varying from 80% to 90% [27], [28]); (3) people who misuse alcohol take up these services on a much wider scale than the available brief, low-intensity face-to-face interventions in primary care settings [24], [29]; and (4) people differ in whether they desire additional help from professionals [28]. Studies have also shown such unguided interventions to be effective in reducing adult alcohol misuse as compared to no-intervention control conditions. A 2011 meta-analysis by Riper and colleagues [26] found a small but significant effect size of *g* = 0.39 (95% CI: 0.23–0.56) for unguided self-help interventions via the Internet. Numbers needed to treat (NNT = 5) were comparable to those for face-to-face brief interventions in primary care settings [30]. Small effect sizes also emerged in meta-analyses on various health promotion interventions, including alcohol use [31], [32]. Few cost-effectiveness studies are available, but they indicate potential economic gain from Internet-based interventions for adult alcohol misuse [33].

The number of eligible randomised controlled trials on web-based interventions for alcohol misuse was rather limited in these systematic reviews and meta-analyses. The Riper meta-analysis [26], for example, identified only 7 eligible randomised controlled trials. This sharply contrasts with meta-analyses assessing the clinical and cost-effectiveness of Internet interventions for depression or anxiety. For example, Richards and Richardson [34] were able to include 19 RCTs on depression in their 2012 meta-analysis. For these disorders, overall evidence is accumulating that guided interventions generally lead to a greater reduction of depressive or anxiety symptoms than unguided interventions [34], [35] and that guided Internet interventions are as effective as face to face interventions [36].

Recently, the numbers of published randomised controlled trials on Internet-based interventions for alcohol misuse has increased, including some that evaluate therapist-led self-help interventions [37], [38]. We therefore decided to conduct this meta-analysis. We investigated the overall effectiveness of alcohol interventions in comparison to no-intervention controls and, if possible in terms of alcohol consumption reduction, drinking within the guidelines for low risk drinking and actual amounts reduced. We then examined whether certain study characteristics, such as the guided or unguided design of interventions, affected the primary outcome measure of alcohol consumption. To the best of our knowledge, this is the first meta-analysis that includes both guided and unguided Internet interventions to address problematic alcohol consumption among adults.

## Methods

### Identification of Studies

We conducted literature searches up to September 2013 in the following bibliographic databases: MEDLINE, PsycINFO, Science Citation Index Expanded, Social Sciences Citation Index, Arts and Humanities Citation Index, CINAHL, PUBMED and EMBASE, using key words and text words. Words indicating online interventions (Internet, Web, online, computer, mobile) were combined with terms indicative of type of treatment (self-help, brief intervention, treatment, unguided, guided, supported, low-intensity) and problematic alcohol use (alcohol abuse, alcohol misuse, problem drinking, hazardous, harmful, dependence, abstinence). We also re-examined exclusion lists of papers retrieved for our previous meta-analysis [26] to see if any would meet the inclusion criteria for the current study. No language restrictions were applied.

**Figure 1:**
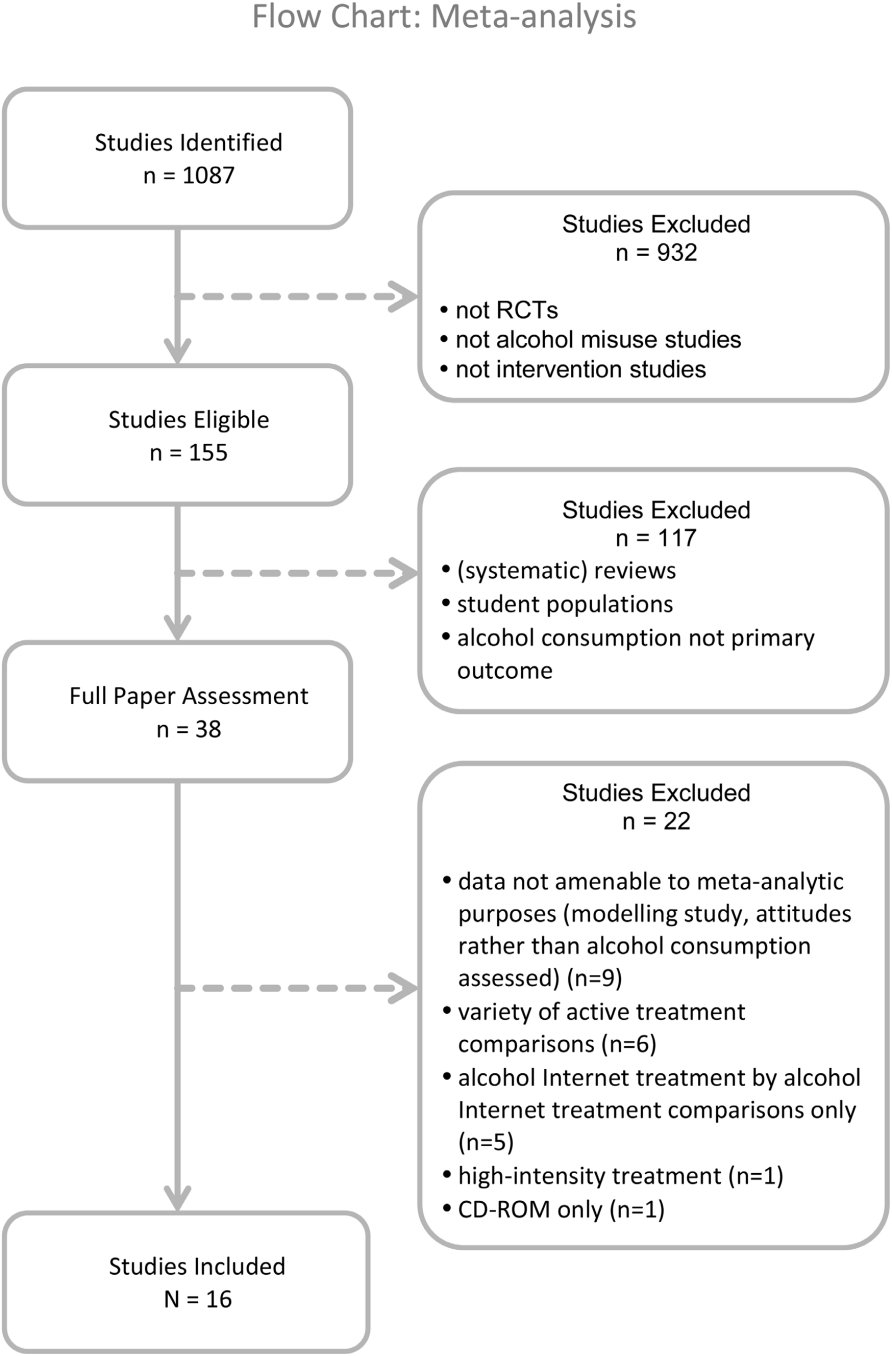
Study inclusion meta-analysis: flow chart.

Our initial selection was based on titles and abstracts. If these yielded insufficient information to assess the eligibility criteria, full-text articles were retrieved and assessed in terms of our inclusion criteria. All papers included or excluded at all stages were assessed by two independent raters (authors HR and HH) (see figure 1). The same raters assessed the effect sizes and moderator variables in the included studies. Any disagreement was resolved by discussion. A protocol does not exist for this meta-analysis; steps undertaken are described in this method section.

### Eligibility Criteria

Randomised controlled trials were included that (1) compared a web-based intervention with a control group (in an assessment-only, waitlisted or alcohol information brochure control condition); (2) included a low-intensity self-help intervention that the participant could perform on a computer or mobile phone, with or without guidance from a professional; (3) assessed alcohol drinking behaviour in terms of quantity consumed as a primary outcome measure; (4) studied adults aged 18 or older; (5) included alcohol drinkers who exceeded local guidelines for low-risk drinking (if lower-risk participants were also included, we retained only the results of those with alcohol misuse).

### Risk-of-bias Assessment and Data Extraction

We assessed the validity of the included studies on the basis of four criteria from the risk-of-bias assessment tool developed by the Cochrane Collaboration [39]. It verifies study attributes in randomised controlled trials that are potential sources of bias, including (1) adequacy of allocation sequence generation, (2) concealment of the allocation to the different conditions, (3) blinding of assessors and outcomes and (4) handling of incomplete outcome data using intention-to-treat analyses (this was rated as positive if intention-to-treat analyses were performed, thus retaining all randomised participants in the analyses; see table 1). Authors HH and MB assessed risk of bias as Yes, No or Unclear using the Cochrane Collaboration tool (see 2.3).

**Table 1:** Study characteristics of 16 randomised controlled trials of Internet-based interventions for adult alcohol misuse.

Author, year, country	Recruitment	Target group,inclusion criteria	Deliverymode |setting	Type of support |intervention(s) | dosage	Comparison (interventionvs control)	N^a^/N^b^	Outcomemeasure	ITT or CO	Post-treat­mentassessment	DO	Risk of bias^1–4^	Baseline: M (SD) drinks^k^ per week | and/or AUDIT score
Araki, 2006, Japan	Workplace	Males, GGT ≥60 IU/I	E-mail | workplace	Guided |HE | 2 sessions	Guided vs AO	12/12	Drinks per day	CO	2 months	3%	1) Unclear* 2) No 3) Unclear* 4) No	Drinks: 18.6 (12.2) | AUDIT: -
Bischof, 2008, Germany	General practice	GP patients age 18–64, alcohol dependence or abuse, or >20/30 g of alcohol per day for females/males in past 4 weeks, or >60/80 g for females/males on >1 occasion in the past 4 weeks	E-mail, phone | home	Guided |BCC, MI, TTM | 4 sessions (FC) or max. 3 sessions (SC)	1) Guided SC^c^ vs AB/H; 2) Guided FC^d^ vs AB/H	1) 131/139∧; 2) 138/139∧	Grams of alcohol per day	ITT	12 months	8%	1) Yes 2) No 3) Yes 4) Yes	Drinks: 31.9 (35.2) | AUDIT: -
Blankers, 2011, Netherlands	Community	Males/females, AUDIT≥8 and ≥14 units/week	Internet | home	Guided and unguided |CBT, MI | 7 sessions guided, 6 modules unguided	1) Guided vs WL; 2) Unguided vs WL	1) 68/69∧; 2) 68/69∧	Drinks in past week	ITT	3 months; 6 months	30%	1) Yes 2) No 3) Yes 4) Yes	Drinks: 44.7 (26.4) | AUDIT: 19.5 (5.1)
Boon & Huiberts, 2006, Netherlands	Community	Males/females, ≥21/14 units/week; or ≥6/4 units on ≥1 day/week	Internet | research setting	Unguided |PNF | single-session	Unguided vs AB	102/89	Drinks per week	CO	9 months	32%	1) Yes 2) No 3) Yes 4) No	Drinks: **-** | AUDIT: -
Boon, 2011, Netherlands	Community	Males, ≥21 units/week or ≥6 units ≥1 day per week	Internet | research setting	Unguided |PNF | single-session	Unguided vs AB	230/220	Drinks per week	ITT	1 month; 6 months	8%	1) Yes 2) Yes 3) Yes 4) Yes	Drinks: 31.3 (16.8) | AUDIT: -
Brendryen, 2013, Norway	Community	FAST ≥3	Internet | home	Unguided | PNF, CBT | multiple sessions	Unguided vs AB	125/119	Drinks per week	ITT	2 months; 6 months*	37%	1) Yes 2) Yes 3) Yes 4) Yes	Drinks: 23.5 (16.1) | AUDIT: -
Cunningham, 2009, Canada	Community	Males/females, AUDIT ≥11	Internet | home	Unguided |PNF | single-session	Unguided vs AB	35/37	Drinks per week	ITT	3 months; 6 months	8%	1) Yes 2) No 3) Yes 4) Yes	Drinks: 22.5 (12.6) | AUDIT-C: 8.9-
Delrahim-Howlett, 2011, USA	Women’s and infant services	Females, ≥3 units on ≥1 day/month	Internet | health care	Unguided |PNF | single-session	Unguided vs ABdouble-blind	68/67	Drinks per occasion	CO	1 month; 2 months	10%	1) Yes 2) Yes 3) Yes 4) No	Drinks: 8.2 (7.5) | AUDIT: -
Doumas & Hannah, 2008, USA	Workplace	Males/females^&^, ≥1 occasions with ≥5/4 units/past 2 weeks	Internet | workplace	Guided and unguided |PNF, MI | single-session	1) Guided vs AO 2) Unguided vs AO	1)12/16; 2) 8/7^ l^	Drinks per week	CO	1 month	37%	1) Yes 2) No 3) Yes 4) No	Drinks: 3.89 (2.44) | AUDIT: -
Hansen, 2012, Denmark	Community	Males/females, ≥21/14 units/week	Internet | home	Unguided |PNF | single-session	1) Unguided advice vs AO 2) Unguided PF^f^ vs AO	1) 476/454; 2) 450/454	Drinks per week	ITT	6 months; 12 months	37%	1) Yes 2) No 3) Yes 4) Yes	Drinks: 32.8 (13.5) | AUDIT: -
Hester, 2005, USA	Community	Males/females, AUDIT ≥8	CD-ROM | health care	Unguided |PNF, BSC, MI |single-session extended	Unguided vs WL	35/26	Drinks per day	CO	4 weeks	0%	1) Yes 2) No 3) Yes 4) No	Drinks: 47.6 (42.9) | AUDIT: 19.7 (-)
Pemberton^m^, 2011, USA	Military	Males/females^&^,/all interested (we assessed high-risk group only)	Internet | home	Unguided |HBM, MI, SCM | 3 modules	1) Unguided combined vs AO; 2) Unguided MI^h^ vs AO	1) 144/72; 2) 95/73^ l^	Drinks per occasion	ITT	1 month		1) No 2) NA* 3) Yes 4) Yes	Drinks: - | AUDIT: -
Riper, 2007, Netherlands	Community	Males/females, >21/14 units/week; or ≥6/4 units ≥1 day for past 3 months	Internet | home	Unguided |BSC, CBT, MI |6 weeks	Unguided vs AB	130/131	Drinks per week	ITT	6 months	42%	1) Yes 2) No 3) Yes 4) Yes	Drinks: 43.6 (21.7) | AUDIT: -
Schulz, 2013, Netherlands	Community/online access panel	AUDIT; (1) >1 drink (females) or >2 drinks (males) per day, (2) drinking more than 5 days per week; (3) >7 AUDIT	Internet | home	PNF | 3 sessions	1) Alternating vs AO 2) Summative vs AO	1) 132/135∧; 2) 181/135∧	Mean drinks per week	ITT	6 months		1) Yes 2) Yes 3) Yes 4) Yes	Drinks: 12.9 (11.2) | AUDIT: -
Suffoletto, 2012, USA	Emergency department	Males/females, AUDIT-C ≥4/3	SMS | home	Unguided |PNF | 12 weeks	1) Unguided^i^ vs AO; 2) Unguided^j^ vs AO	1) 14/13∧; 2) 12/13∧	Drinks per drinking day	CO	3 months	13%	1) Yes 2) Yes 3) Yes 4) No	Drinks: - | AUDIT: -
Wallace, 2011, UK	Alcohol website	Males/females, AUDIT-C ≥5	Internet | home	Unguided |CBT | 3 modules	Unguided vs CW	582/695	Drinks in past week	ITT	3 months	45%	1) Yes 2) Yes 3) Yes 4) Yes	36.8 (25.0) | AUDIT: 18.8 (7.3)

*Notes:* ^1–4^: Risk of bias: 1): adequate sequence generation; 2): report of conditions by an independent party; 3): blinding of outcome assessor or using self-report outcomes only; 4): intention to treat analyses; *: in regression analysis coded as NO; ∧ control group reported twice; &: at risk group drinkers only; **^a^**intervention group size; control group size; ^c^full-care intervention; ?stepped-care intervention; ^e^brief personalised feedback intervention; ^f^brief personalised advice intervention;^ g^Alcohol Savvy intervention; ^h^Drinker’s Check-Up intervention; ^i^text message feedback intervention; ^j^text message assessment intervention; ^k^1 ‘drink’ contains 10 grams of ethanol; ^l^only a subsample included;^ m^not all participants randomly allocated; AB alcohol brochure control; AB/H alcohol/health brochure control; AO assessment-only control; AUDIT: Alcohol Use Disorders Identification Test; AUDI-C ^brief AUDIT, composite^ measure that consists of respondents’ scores on frequency of drinking, drinks per drinking day and frequency of five or more drinks on one occasion; BSC behavioural self-control training; CBT cognitive-behavioural therapy; CO completers-only analysis; CW comparator website control; DO Study dropout; FAST Fast Alcohol Screening Test; GGT gamma-glutamyl transpeptidase test; HB Health booklet control; HBM Health Belief model; HE health education; ITT intention-to-treat analysis; MI motivational interviewing; PFGS personalised feedback with goal setting; PNF personalised normative feedback; SCM stages of change model; TTM transtheoretical model of change.

### Study Characteristics

Characteristics of the analysed studies are described in table 1. We coded (1) year of study and country of origin; (2) participant characteristics: alcohol consumption level at baseline, recruitment setting, target group; (3) intervention characteristics: therapeutic principles, mode and setting of delivery, guided or unguided self-help format, number of sessions; and (4) other study characteristics: type of control condition, number of participants in each comparative condition, primary outcome measure (how consumed quantities of alcohol were assessed), intention-to-treat (ITT) versus completers-only (CO) analyses, post-treatment assessments in months, dropout rate and risk of bias. The brief self-help alcohol interventions we identified were based on one or more of the following principles: behavioural self-control [40], motivational interviewing [41], transtheoretical model of change [42], cognitive-behavioural therapy (CBT, [43]), and personalised normative feedback (PNF [44]).

### Meta-analyses

We first calculated a mean alcohol consumption effect size for each comparison between an alcohol intervention and a control group. Effect sizes were calculated by subtracting the average post-test score of the alcohol self-help group from the average score of the comparison group and dividing the result by the pooled standard deviations of the two groups. This effect size is known as Cohen’s *d*. As the effect size *d* is subject to small-sample bias, it can be adjusted by using a scaling factor, which is multiplied by *d* to arrive at Hedges’s bias-corrected effect size *g*, so that g = d (1−[3/4(n_1_+n_0_]−9). An effect size of 0.50 indicates that the mean of the experimental group is half a standard deviation larger than the mean of the control group. Effect sizes of approximately 0.80 can be considered large, 0.5 as moderate, and 0.2 small [45]. Participant adherence to low-risk alcohol guidelines (by either abstaining or not exceeding recommended limits) was assessed in terms of percentages (yes/no).

If means and standard deviations were not reported, we contacted the study authors to obtain these and/or we used the procedures of the Comprehensive Meta-Analysis software (CMA, version 2.2.021) to calculate the effect size using continuous or dichotomous outcomes. In the event these were not available either, we used other statistics (such a *t*- or *p*-value). Where possible, data from intention-to-treat analyses were used; completers-only data were used if the former were unavailable. If more than one alcohol consumption outcome measure was reported in a single study, we averaged the effect sizes from those measures to produce a single summary effect size for use in the meta-analysis, statistically adjusting those calculations to account for variance introduced by the multiple measures [46].

We calculated the mean effect sizes using a random effects model. This assumes that the included studies were drawn from ‘populations’ of studies that systematically differed from one another (heterogeneity). As a test of homogeneity of effect sizes, we calculated the *I^2^* statistic, an indicator of heterogeneity in percentages. A value of 0% indicates no observed heterogeneity, and larger values show increasing heterogeneity, with 25% as low, 50% as moderate and 75% as high [47]. As the *I^2^* statistic is known to be imprecise [48], we calculated the 95% confidence interval using the non-central chi-squared-based approach within the heterogi module in Stata [49]. We also estimated numbers needed to treat (NNTs) [50].

#### Subgroup analyses

In consideration of the literature, ten subgroups analyses were carried out using the mixed effects model, whereby studies within subgroups are pooled with the random effects model (see table 2). Tests for significant differences between subgroups are then performed with the fixed effects model. For continuous variables, we used bivariate meta-regression analyses to test whether there was a significant relationship between each variable and the alcohol effect size, as indicated by a *Z-*value and an associated *p*-value.

**Table 2:** Effects of low-intensity Internet alcohol treatment on alcohol consumption in comparison with no-intervention controls, and subgroup analyses of associations between effect sizes and study characteristics (Hedges’s *g*).

Experimental/Control	subgroup	n comparisons	*g*	95% CI	*I^2^* ^b^	95% CI	*P* ^d^	NNT^e^
***All studies*** **^a^**		23	0.20	0.13–0.27***	27	0∼56		8.93
One effect size per study (lowest excluded)		16	0.23	0.15–0.32***	34	0∼64		7.69
One effect size per study (highest excluded)		16	0.20	0.10–0.29***	39*	0∼66		8.93
Lowest effect size removed		22	0.20	0.13–0.27***	24	0∼55		8.93
Highest effect size removed		22	0.19	0.12–0.25***	20	0∼53		9.43
***Subgroup analyses (N = 23)***								
Type of control	Assessment-only	11	0.15	0.06–0.24***	0	0∼60	**.056**	11.90
	Waitlist	3	0.48	0.22–0.73***	0	0∼90		3.76
	Alcohol brochure	9	0.20	0.08–0.31***	48	0∼76		8.93
Blinding of participants	Yes	8	0.16	0.04–0.28**	30	0∼69	.393	11.11
	No	15	0.23	0.14–0.32***	23	0∼58		7.69
Analyses	ITT	13	0.22	0.13–0.31***	30	0∼64	.600	8.06
	CO	10	0.18	0.05–0.30**	19	0∼60		9.80
Recruitment	Community	11	0.21	0.11–0.31***	52*	4∼76	.962	8.47
	Primary care/clinic	7	0.21	0.04–0.39**	24	0∼66		8.47
	Work	5	0.24	0.05–0.412**	0	0∼71		7.46
Population for inclusion	At-risk drinking cut-off	12	0.19	0.09–0.28***	40	0∼70	.530	9.43
	AUDIT (10)/(FAST (1)	11	0.24	0.12–0.35***	1	0∼61		7.46
Focus of treatment	PNF	9	0.16	0.07–0.24***	0	0∼65	.236	11.11
	Combined	14	0.24	0.13–0.35***	42*	0∼69		7.46
Professional guidance	Yes	5	0.23	0.05–0.41**	10	0∼81	.730	7.69
	No	18	0.20	0.12–0.28***	33	43∼87		8.93
Number of sessions	Single	8	0.16	0.08–0.25***	0	0∼68	.399	11.11
	More than one	15	0.22	0.12–0.33***	42*	0∼68		8.06
Gender	Male only	4	0.26	0.12–0.40***	0	0∼85	.484	8.06
	Male and female	19	0.20	0.11–0.28***	35	0∼63		8.93

*Notes:* **bold** – near significance;^ a^-1 according to the random effects model;^ a^-2: according to the mixed effects model ^b^The P-values in this column indicatewhether the Q-statistic is significant (I2-statistics do not include a test of significance).; ^c^The P-values in this column indicate whether the difference between the effect sizes in the subgroups is significant.; *P≤0.05; **P<0.01; ***P≤0.001; AUDIT: Alcohol Use Disorders Identification Test; CBT cognitive-behavioural therapy; CI = confidence interval; CO completers-only analysis; DSM = Diagnostic Statistical Manual of Mental Disorders; FAST: Fast Alcohol Screening Test; ITT intention-to-treat analysis; MI motivational interviewing; n comp = number of comparisons; NNT = number needed to treat; PNF personalised normative feedback.

### Power Calculations

We calculated both beforehand and afterwards how many studies would be needed to ensure sufficient statistical power to identify relevant effects as we expected a small to moderate effect size [26], [51]. The power calculations were carried out according to the procedures described by Borenstein and colleagues [46]. Beforehand we hoped to find enough studies to enable identification of a small effect size of *d* = 0.40 based on the random effects model. The power calculations indicated that this would require at least ten studies with a mean sample size of 70 participants per condition. That conservatively assumes a medium level of between-study variance (τ^2^), a statistical power of .80 and a significance level of α<.05.

### Publication Bias

To detect possible publication bias, we visually examined the funnel plots of the primary outcome measures for symmetry. We conducted Egger’s linear regression test of the intercept to quantify the bias captured by the funnel plot and test whether it was significant [52]. Duval and Tweedie trim-and-fill procedure was performed to further verify whether the pooled effect size estimate was unbiased [53]. These procedures were all performed with CMA.

## Results

### Selection and Inclusion of Studies

A flowchart depicting our study selection procedure is shown in figure 1. In reporting the results we followed the guidelines of the PRISMA statement (see Checklist S1) [54].

### Characteristics of Included Studies

Table 1 summarises the selected characteristics of the 16 studies (containing 23 comparisons) included in the meta-analysis. The studies assessed a total of 5,612 participants (3,268 in experimental and 2,344 in control conditions) and thus provided sufficient statistical power to detect a small effect size (see section 2.6).

In five studies, sample inclusion was based on self-reported alcohol consumption, using cut-off points indicative of alcohol use exceeding low-risk guidelines, with differing levels for men and women. Five other studies applied the Alcohol Use Identification Test (AUDIT [55]) or the Fast Alcohol Screening Test (FAST [56]). Two studies applied both AUDIT and alcohol consumption cut-off points; one selected on the basis of GGT (gamma-glutamyl transpeptidase) testing. Two of the sixteen studies [57], [58] included anyone interested in participating; for these studies, we confined our analysis to subgroups belonging to the at-risk drinking population.

Seven studies applied a single-focus therapeutic strategy, which in six cases was personalised normative feedback (PNF) and in one case a generic health education approach. The other nine studies used combined treatment approaches consisting of motivational interviewing (MI), personalised normative feedback (PNF), cognitive-behavioural therapy (CBT) and/or behavioural self-control and change principles. The control groups represented assessment-only (*n* = 6), waitlisted (*n* = 3) or alcohol information brochure conditions (*n* = 7). The outcome assessments to indicate alcohol consumption were expressed in terms of mean standard drinks per drinking day, total numbers of standard drinks in the previous week, mean standard drinks per drinking occasion or amounts of alcohol in grams.

The risk of bias varied among the studies (table 1), with 14 studies reporting adequate sequence generation, 6 reporting allocation to conditions by an independent party, 15 reporting blinding of outcome assessors or using self-report outcomes only, and 10 using intention-to-treat analyses. Dropout rates varied from 0% to 42%.

### Meta-analysis

**Figure 2:**
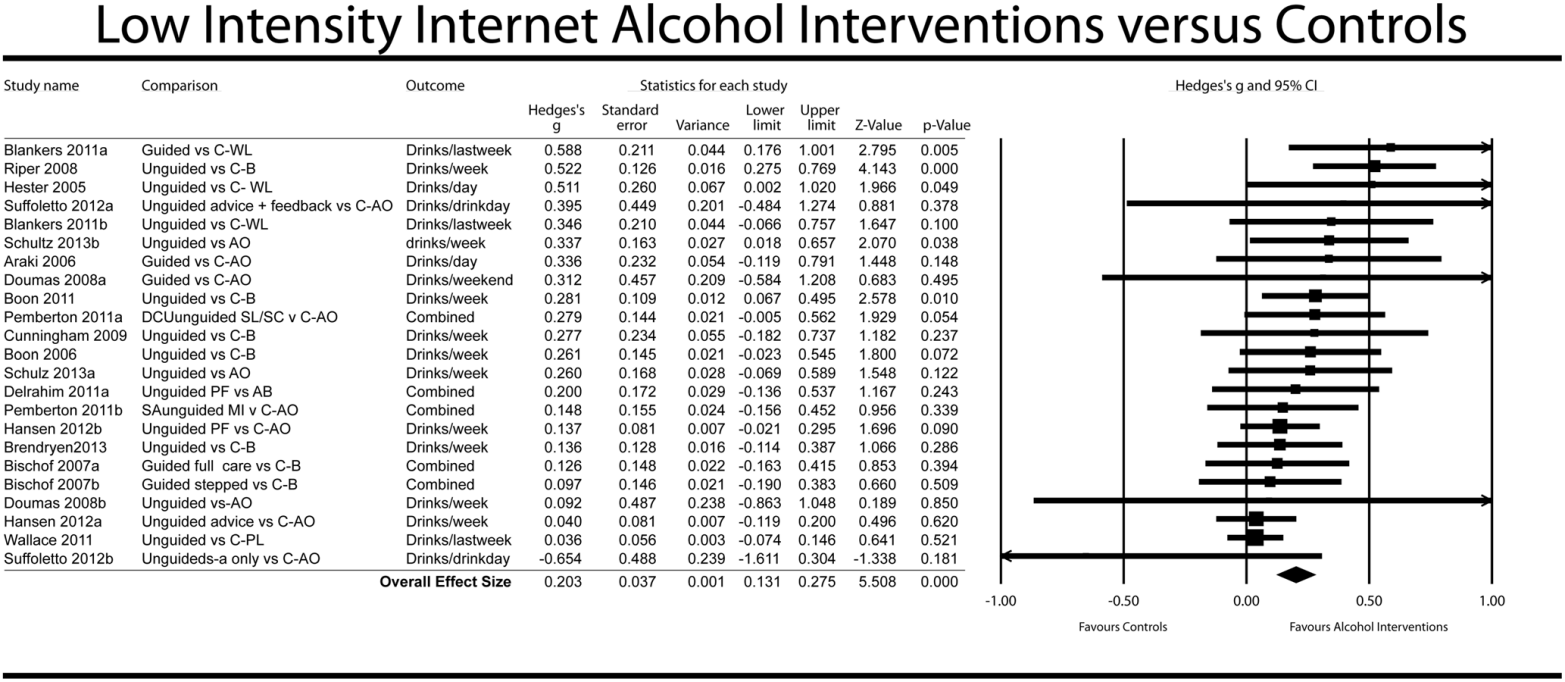
Results of meta-analysis: forest plot.

The effect of low-intensity Internet-based alcohol interventions to reduce alcohol consumption in comparison to controls was small but significant at post-test (*g* = 0.20, random effects model, 95% CI: 0.13–0.27, *p*<.001, NNT = 8.93). Results are shown in figure 2 (from high to low study effect sizes) and table 2. Heterogeneity was low but with a moderate confidence interval (*I^2^* = 27, 95% CI: 0–56). A post-hoc power calculation showed that our set of studies had sufficient statistical power (0.99) on the basis of the random effects model (with a low level of between-study variance, τ^2^ = .001, and a significance level of α <.05).

Seven studies [37], [57]–[62] compared more than one group receiving differing types of Internet-based brief treatment with a single control group, so that our analysis included multiple comparisons from these studies. The fact that the comparisons were not independent of one another could have artificially reduced the heterogeneity of the analysed studies, thereby affecting the pooled effect size. To test this, we carried out sensitivity analyses that included only one effect size per study. As table 2 shows, removal of the comparisons with the highest effect sizes had almost no influence on the pooled effect size nor did removing the comparisons with the lowest effect sizes. Removal of the one guided intervention comparison with the highest effect size (in Blankers [37]) did not influence the observed effect size either, nor did removal of that with the lowest effect size [62].

### Mean Alcohol Consumption Reduction and Drinking within Low Risk Guidelines

On average, the intervention participants were drinking 2,2 alcohol consumptions (22 grams of ethanol) less per week at post-treatment than controls (*n* = 14; 95% CI: 0.87–3.46, *p* = .001, I^2^ = 0, *p*<.001; mean difference). Post-test differences [37], [61], [63]–[65]) revealed that intervention participants were significantly more likely than controls to have reduced their alcohol consumption to within the low-risk guidelines as compared to controls (*n* = 6; RD 0.13, 95% CI: 0.09–0.17, *p*<.001, I^2^ = 0, non-significant).

### Subgroup Analyses

Subgroup analyses revealed no significant differences between study characteristics and effect sizes for alcohol consumption (see table 2). The differences between types of control conditions bordered on significance (*p* = .056). We found a higher effect size for decreased alcohol consumption for studies applying a waitlist control condition (*g* = 0.48) than those applying an alcohol brochure condition (*g* = 0.20) or an assessment-only condition (*g* = 0.15).

### Follow-up Assessments

No significant differences in effect remained at follow-up (6 to 12 months after baseline) between the six unguided interventions (8 comparisons) and control groups (*g* = 0.06, 95% CI: −0.14–0.25, *p* = .567, random effects model) for which such follow-up assessments were available. No follow-up assessments were available for the guided interventions.

### Regression Analyses

Meta-regression analyses with Hedges’s *g* as the dependent variable uncovered no significant associations between effect size and either the number of alcohol intervention sessions (β = −0.0001, 95% CI: −0.004–0.003, *p* = .938) or the risk of bias (β = 0.068, 95% CI: −0.15–0.01, *p* = .089) in the studies assessed.

### Publication Bias

There was evidence of publication bias in terms of Duval and Tweedie’s trim-and-fill method. After adjustment for missing studies, the effect size for decreased alcohol consumption diminished from *g* = 0.20 to *g* = 0.14 (95% CI: 0.06–0.22; trimmed studies *n* = 8, random effects model). Egger’s test indicated an asymmetric funnel plot (*p* = .028, two-tailed).

## Discussion

This meta-analysis showed a small but significant overall effect size (*g* = 0.20) in favour of low-intensity Internet-based self-help interventions to curb alcohol misuse over control conditions. At post-treatment, intervention participants were consuming an average of 22 grams of alcohol less per week than controls. The reduction appears lower than those found in analyses of face-to-face brief interventions in primary care. The latter include a Cochrane Collaboration systematic review of 29 primary care trials [30], which reported a significantly reduced weekly consumption of 38 grams at one-year follow-up (see also the study by Jonas and colleagues [15]). In our study, Internet intervention participants were 13% more likely than controls to stay within the guidelines after treatment (*n* = 6). This is in line with the study of Jonas and colleagues [15] who showed that those receiving primary care alcohol interventions were 11% more likely to do so when compared with controls.

The overall effect size in this meta-analysis is lower than, the effect size found in our previous study that focused solely on *unguided* e-interventions (7 studies, *g* = 0.39) [26]. Some explanations for this may lie in the three-times-higher number of comparisons involved in the current analysis and the fact that studies with null findings were now included [60], [66]. The current overall effect also compares with meta-analyses of face-to-face primary care samples [3], [30], [67], [68] and of non-clinical samples performing postal self-help interventions based on bibliotherapy [69]–[71]. These studies all showed significant small to moderate effect sizes for curbing alcohol misuse. We found no significant differences between experimental and control conditions for those studies that assessed follow-up outcomes up to twelve months. Such a decay of intervention effects is not uncommon; larger effect sizes are generally found at the earliest follow-ups [15], [68]. Some studies of brief interventions for problem drinking have nonetheless reported positive influences on alcohol reduction up to four years later [67] and on mortality [72].

### Subgroup Analyses

Results of the subgroup analyses did not reveal any significant differences between experimental and control conditions. All these results need to be interpreted with caution, as the number of studies included in the subgroup analyses was low. This means that there where either no real differences between these conditions or that real difference may have gone undetected. The variation in types of control predictors was of borderline significance (*p* = .056) in terms of effect. A moderate effect size was found for studies using waitlist control conditions, while those with assessment-only or alcohol brochure control showed small ones. Other studies have similarly reported higher effect sizes for intervention studies that used waitlisted groups as comparators [73]. One explanation could be that waitlisted participants tend to delay behavioural change because they anticipate professional help in the near future; that could cause overestimation of the intervention effect.

We did not find a significantly greater effect size for guided (*g* = 0.23) than for unguided (*g* = 0.20) alcohol interventions, even though such significant differences in benefit have been overwhelmingly established for web-based treatment of depression and anxiety [34], [74]. Our lack of significant effect might be due to the fact that many participants in web-based alcohol interventions are first-time help seekers; they may hence derive health gains from their first formal attempt at behavioural change, whether unguided or guided. Alternatively, a real difference may have gone undetected as a consequence of the low number of guided interventions in our analysis (5 comparisons. Only the Blankers and colleagues [37], and the Doumas and Hannah [57] studies provided direct comparisons between guided and unguided interventions. Both showed a small, but non significant, difference in effect in terms of alcohol consumption reduction in favour of guided interventions. These non-significant results are probably due to lack of power in both studies as well.

In contrast with our previous analysis from 2011 [26], we now found no significant differences in alcohol reduction between single-session interventions and more extended ones. Although the literature is somewhat inconclusive [75], it provides some confirmation for our current findings [30], [68], [76].

Web-based interventions may offer considerable promise for reducing alcohol misuse by women. We found no difference in alcohol consumption outcomes between all-male and mixed-gender samples, whereas several studies on face-to-face alcohol interventions in primary care had reported that men were more likely to benefit than women [30], [67]. In the case of Internet-based interventions for alcohol misuse, however, female uptake appears equally as high as male uptake [28], [77] and have been seen to have similar impacts on female and on male drinking, or possibly even greater influence on females, as suggested by Riper and colleagues (2008, [78]).

No difference in effect sizes emerged between studies that used cut-off points in alcohol consumption as study inclusion criteria and those that applied stricter measurements such as AUDIT [55]. AUDIT scores among the latter were high (averaging around 20, indicating a potentially high risk of alcohol dependence amongst participants. Internet interventions might thus well be effective for a broad spectrum of people who misuse alcohol. Just as in many trials of brief alcohol interventions and routine practice studies [18], [79], the studies in our analysis did not conduct diagnostic interviews to assess for alcohol dependence. This lack of actual diagnoses also hampers any attempt to use the available data to test the common view that self-help interventions are particularly suited to people with less serious alcohol problems, but less appropriate for those with alcohol dependence [3], [80].

Several limitations underlie our meta-analysis that may have affected our overall results. First, as indicated the number of studies included in the subgroup analyses was low. The resulting insufficient statistical power may call into question our outcomes whereby no significant difference between a number of conditions could be observed. Second, some included studies had substantial dropout rates (above 30%; see table 1). High study (and treatment) dropout rates are a common phenomenon in both online and offline self-help interventions for alcohol misuse and in Internet interventions in general [81], [82]. Third, the impact of online self-help in reducing alcohol consumption may have been underestimated here because control group drinking was also assessed; such assessment alone possibly motivates controls to reduce their consumption [83], [84]. Last but not least, we were not able to evaluate possible negative side-effects of Internet-based interventions for problem drinking, as the studies lack reports on such effects.

### Clinical Implications

The results of this study support the use of guided and unguided Internet-based self-help interventions for curbing alcohol misuse in various settings (primary health care, work environments and the community). Although the overall effect size for these interventions was small, the public health impact could be substantial if large numbers of people who misuse alcohol were to take part in these interventions. Decreasing alcohol consumption as well as abstinence and adherence to low-risk guidelines are health benefits in their own right and potential predictors of longer-term maintenance of decreased alcohol consumption [85]. A modelling study by Smit and colleagues [86] has suggested that if online interventions were to partially replace conventional face-to-face brief alcohol interventions, that could sustain comparable levels of population health at lower costs.

### Future Research

Future studies should try to assess which type (s) of alcohol misuse populations could benefit most from brief online alcohol interventions. More studies with longer-term follow-up periods (over 12 months) are needed, too, to assess the maintenance of intervention effects over time in terms of reduced consumption and other health outcomes. Studies focusing on the effects of guided self-help interventions in curbing alcohol misuse, in direct comparison with unguided interventions, could shed light on whether the former produce better outcomes and, if so, at what costs. It is also important to investigate whether combining low-intensity Internet interventions with brief face-to-face interventions (blending of channels) would increase the effectiveness of interventions to curb alcohol misuse.

## Conclusion

Internet interventions are capable of reaching out effectively to the large group of people who engage in alcohol misuse. From a public health perspective, this justifies an upscaling of such interventions in routine practice as well as in a wide range of community settings.

## Supporting Information

Checklist S1 PRISMA checklist.
(DOC)
